# Skin Temperature in Master Long-Distance Runners—Results From a Field Study at the 2018 World Master Athletics Championships

**DOI:** 10.3389/fspor.2020.00031

**Published:** 2020-04-09

**Authors:** Bergita Ganse, Hans Degens

**Affiliations:** ^1^Department of Life Sciences, Research Centre for Musculoskeletal Science & Sports Medicine, Manchester Metropolitan University, Manchester, United Kingdom; ^2^Institute of Sport Science and Innovations, Lithuanian Sports University, Kaunas, Lithuania

**Keywords:** running, thermography, heat, sweating, aging, skin temperature, thermal physiology, endurance

## Abstract

Older people and athletes show impairments in thermoregulation, but this has not yet been studied during a running competition. The aim of the study was to assess (1) whether there are age-related differences in skin temperature during the last stage of a race in well-trained master athletes and (2) to what extent such differences are related to running speed and sex. To investigate this, we used thermography to measure maximum skin temperatures of the head, legs and hands of participants of the 2018 World Master Athletics (WMA) Championships when they were approximately 9,600 m into a 10,000-m road race. Of the 813 runners, 404 were analyzed (142 women, 262 men) including athletes of age groups 35 to 85. All ≥70-year-old athletes completed the race; all 16 non-finishers were younger. The hand temperature was lower than that of the head and legs (*p* < 0.001). Stepwise regression revealed that head ($R_{\text{adj}}^{2}$ = 0.143; *p* < 0.001) and hand temperature decreased with increasing speed ($R_{\text{adj}}^{2}$ = 0.092; *p* < 0.001). Sex was the most important determinant of leg skin temperature ($R_{\text{adj}}^{2}$ = 0.054; *p* < 0.001), men having higher leg temperatures than women, with a small negative contribution of speed ($R_{\text{adj}}^{2}$ increased to 0.069). In conclusion, higher running speed is associated with lower skin temperatures, and leg skin temperature is lower in women than men. The absence of an age effect on skin temperature suggests that there is no impairment in heat dissipation in well-trained older athletes.

## Introduction

Old age is associated with impaired thermoregulation and higher risks of heat-related illness and death (Benzinger, 1969; Balmain et al., 2018). During heat waves and in hot environments, thermoregulation by appropriate physiological responses to high temperatures is extremely important, especially in older people and athletes (Patz et al., 2005, 2014; Che Muhamed et al., 2019; Knechtle et al., 2019) and poor thermoregulation may have a negative impact on performance. The significance is illustrated by the observation that just a 1°C higher environmental temperature was associated with a lower performance in the Boston Marathon (Nikolaidis et al., 2019). In the worst case, heat exhaustion or heatstroke may derive from failure to dissipate excessive body heat (Holowatz and Kenney, 2010). Despite the higher risk of heat-related illness in older people, long-distance running has become a popular sport for older athletes (Ganse et al., 2018; Epstein and Yanovich, 2019).

Humans need to maintain their core temperature between 35 and 39°C for optimal functioning of biochemical reactions (Benzinger, 1969). In hot environments, heat is dissipated by radiation, conduction, convection and evaporation. Heat dissipation via these processes is facilitated by sweating, vasodilation of the skin vasculature redistributing blood flow to the skin, and changes in behavior, such as drinking, adapted clothing and searching the shade (Benzinger, 1969; Balmain et al., 2018). The ability to dispose of excess heat—Whole-Body Heat Loss (WBHL)—decreases with age, low cardiorespiratory fitness and high body fat (Gagnon et al., 2013). The impaired WBHL in old age is due to diminished skin vascular responses and sweat gland activity (Kenney and Fowler, 1988; Armstrong and Kenney, 1993; Inoue and Shibasaki, 1996; Holowatz et al., 2010; McGinn et al., 2017). The lower sweat gland activity in turn, may be a consequence of impaired vasodilation of skin vasculature, diminished sensitivity of thermoreceptors and a lower responsiveness of sweat glands to cholinergic stimuli (Sagawa et al., 1988).

Age-related impairments in WBHL are known to be attenuated by regular aerobic exercise training (Inoue et al., 1999; Stapleton et al., 2015a,b), but we expect older athletes to still show some age-related aberrations in skin temperature, particularly in comparison to younger athletes. As little is known about the thermoregulation during running in older athletes, the aim of the study was to measure the skin temperature with thermography in younger and older well-trained athletes in a field study at the World Master Athletics Championships to obtain some first insight about the thermoregulation during a run in older athletes. Infrared camera technology has recently seen significant advances and is now a well-established method in exercise physiology (Hillen et al., 2019).

It was hypothesized that at the end of a 10,000-m road race in moderate temperature (1) older athletes have a higher skin temperature compared to younger athletes, (2) faster athletes have a cooler skin than slower athletes, due to increased heat dissipation by convection, and (3) female athletes have a higher skin temperature than age-matched male athletes.

## Materials and Methods

The Institutional Review Board of RWTH Aachen University Hospital approved the study on October 11, 2017 (reference number EK 300/17) and confirmed that informed consent was not needed.

### Data Collection

Data was collected during the 10,000-m road race event at the 2018 World Master Athletics (WMA) Championships in Malaga, Spain in the morning of 9 September, 2018. The race started at the Ciudad de Malaga Stadium and traveled along the Malaga foreshore, ending in the same stadium. The athletes started in two groups with a time difference of 10 min. The first group comprised men aged 35–69 (start time 9:00 AM) and the second group included women of all ages and the men 70 years and older (start time 9:10 AM).

A FLIR ONE Pro infrared camera (FLIR Systems, Wilsonville, Oregon, USA) with a resolution of 640 × 480 pixels was used to take photos of the athletes on their return into the stadium approximately 9,600 m into the race. During measurements, the camera was standing in the shade. We were unable to obtain data at the beginning of the race, as we could not get close enough to the track in the desired location at the start.

Thermography was chosen over other methods, as it allows to non-invasively measure a great number of athletes in a short time. As the athletes focus on a successful championship participation, they are usually not willing to participate in other types of measurements, such as core temperature or local skin temperature measurements. Athletes have usually spent a great amount of money and preparation time for their participation and do not want to risk their best possible performance.

### Weather Conditions

The weather was without rain, humid (88%) and partly sunny during the race. The temperature increased from 21°C at 9 AM (start) to 23°C at 10:30 AM^1^. As the cut-off time enforced by the local organizers was 1:30 h into the race, the maximum air temperature increase was 2°C. Average wind speed was 8 m·s^−1^ and air pressure was 1,017 mbar.

### Image Analysis

**Figure 1:**
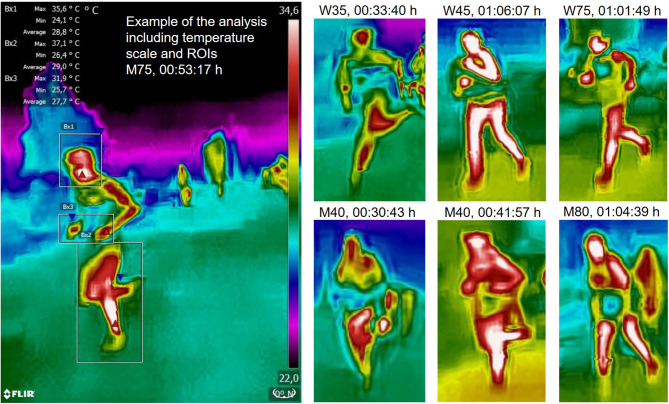
Infrared photos including an example of the analysis and six additional athletes, all in the same temperature-color scale. The FLIR Tools analysis software delivers three temperatures for each ROI: Max., Min., and Average. The maximum was used for analysis in the present study. It can be seen that faster athletes have a lower skin temperature and that athletes show individual patterns in heat distribution.

FLIR Tools version 5.13.18031.2002 was used for image analysis. The FLIR ONE Pro camera takes an infrared and a regular photo at the same time. Athletes were identified using the regular photo by reading their start number. If the number was not readable, publicly available Youtube-videos of the race were used to identify athletes (Youtube videos number xfx06YsSzuo and A9XugO6zPv4). The official result list of the competition^2^ was used to obtain age group, sex and time. The list only includes the age group (5-year steps, example: M35 includes men between 35 and 39 years of age), but not the year of birth. Average running speed was calculated from the time in the results list. Regions of interest (ROIs) were selected around the head, legs and hands of each athlete to assess the maximum skin temperature at each site. ROIs always included the entire body part of interest (Figure 1). Items such as hair, hats, watches and glasses always had a lower temperature than the surrounding skin and therefore did not alter the maximum temperature within the ROI. As the hottest spots were usually located in the middle of the face, the hands and the distal lower leg, we did not experience interference from clothing. As the background was always colder than the athletes' body parts, surrounding objects never altered the measurement. If obstructed by another athlete, a previous or subsequent photo was chosen.

### Statistical Analysis

All statistical tests were performed with IBM^®^ SPSS^®^ Statistics version 25 (IBM corp. Chicago, IL, USA). The figures were made with Microsoft Excel 2019 and SigmaPlot version 14.0. The outcome measures of the study were head, leg and hand skin temperature. An ANOVA was conducted with age and sex as between-subject factors, speed as co-variate and anatomical location as within factor with 3 levels: head, legs and hands. If interactions or an age effect were found additional *post-hoc* Bonferroni-corrected *t*-tests were performed to locate the significant differences. In addition, a stepwise regression was performed to assess the impact of age, sex, and location on the outcome measures, with adjusted *R*^2^-values presented. Significance was assumed at *p* < 0.05. Averages are shown combined with the standard deviation.

## Results

Of the 813 participants (265 women and 548 men) who completed the race, 404 athletes were identified and analyzed (142 women and 262 men) (Table 1). The others could not be identified, in most cases because other runners obstructed the view. Only 12 men and 4 women did not finish the race; all athletes 70 years and older completed the race. Seven of the non-finishers were in age groups 35 and 40. The average speed decreased with increasing age in both men (*R*^2^ = 0.48; *p* < 0.001) and women (*R*^2^ = 0.29; *p* < 0.001).

**Table 1:** Number of athletes in dataset and overall number of finishers.

**Age group**	**Number of men in dataset [*n*]**	**Number of women in dataset [*n*]**	**Total number of athletes in dataset [*n*]**	**Number of male finishers [*n*]**	**Number of female finishers [*n*]**	**Finishers total [*n*]**
35	18	14	32	54	27	81
40	36	20	56	69	40	109
45	32	13	45	75	42	117
50	34	19	53	85	38	123
55	34	24	58	58	40	98
60	41	27	68	83	39	122
65	17	12	29	44	21	65
70	23	6	29	41	10	51
75	15	4	19	23	4	27
80	11	3	14	13	4	17
85	1	0	1	2	0	2
90	0	0	0	1	0	1
Total	262	142	404	548	265	813

**Figure 2:**
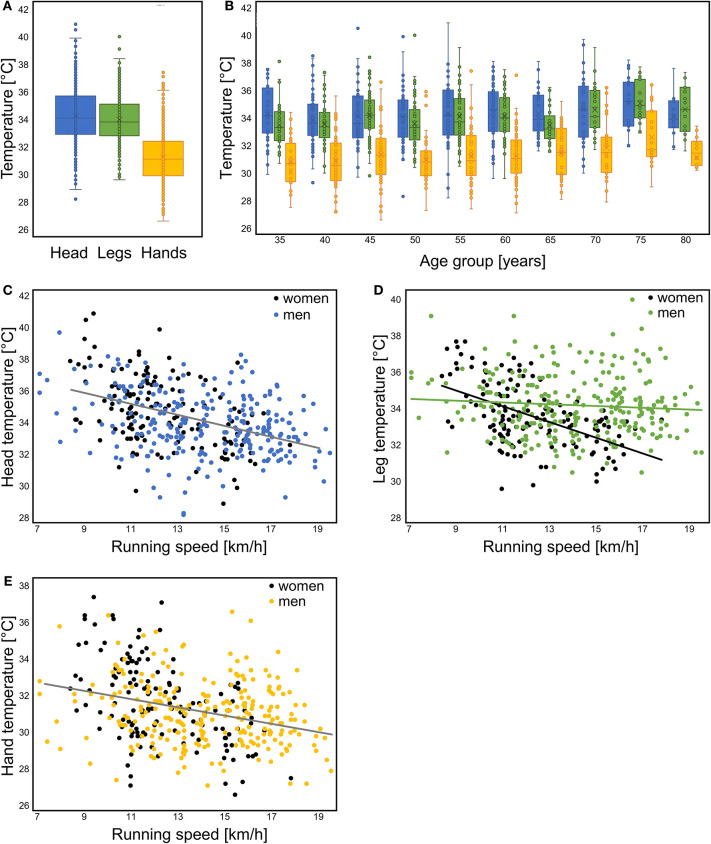
Boxplots showing **(A)** average temperatures for the head, legs and hands, **(B)** the same average values for each age group (except 85, only one participant), **(C–E)** scatter plots with regression lines for speed vs. Temperature. In **(C,E)** only one regression line is shown, as there were no significant sex differences.

**Figure 3:**
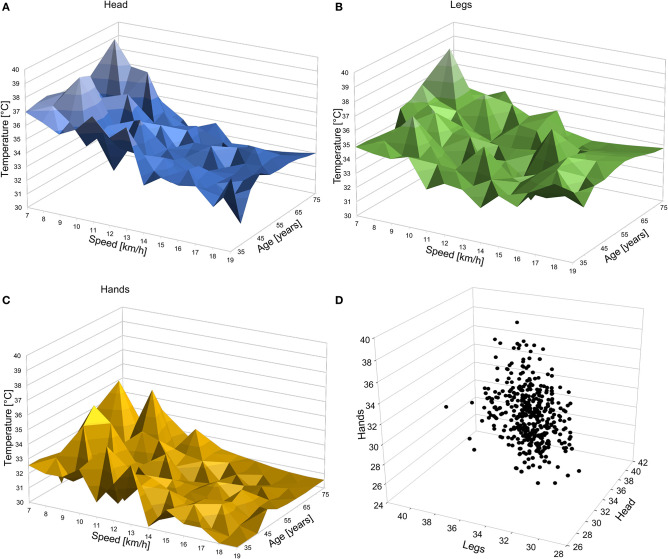
3d plots showing age, speed and maximum skin temperature of the head **(A)**, legs **(B)**, and hands **(C)** of all athletes in the dataset. **(D)** 3d scatter plot of each athlete's head-, leg-, and hand-temperature.

Figure 2A shows that the hand temperature was lower than the temperatures of the legs and head (main effect location: *p* < 0.001), irrespective of age (Figure 2B). Figures 2C–E depict the relationship of running speed with skin temperatures in men and women. Figures 3A–C illustrate the interactions of age and speed for each location. Figure 3D shows the distribution of temperature patterns over participants in a 3d cloud.

The main effects of location (head/legs/hands, *p* < 0.001), age (*p* = 0.008), sex (*p* < 0.001), and speed (*p* < 0.001) on skin temperature were not directly clear as also the following interactions were found: age × sex (*p* = 0.041), age × speed (*p* = 0.009), sex × speed (*p* < 0.001), and location × speed (*p* = 0.001). These interactions mean that the age-related changes in temperature differed between men and women and varied with speed, and that the temperature-differences between men and women and between locations varied with speed, respectively.

To gain more clarity on the determinants of skin temperature we performed stepwise regressions for the head, legs and hands separately, and fed “age,” “sex,” and “speed” as potential factors into the model. It was found that head temperature decreased with increasing speed ($R_{\text{adj}}^{2}$ = 0.143; *p* < 0.001) and a small contribution of age that increased the $R_{\text{adj}}^{2}$ to 0.170 (Figure 2C). Sex was the most important determinant of leg skin temperature ($R_{\text{adj}}^{2}$ = 0.054; *p* < 0.001) with men having a higher leg skin temperature than women (women 33.6 ± 1.7°C, men: 34.2 ± 1.7°C), with a small negative contribution of speed ($R_{\text{adj}}^{2}$ increased to 0.069; Figure 2D). Hand skin temperature also decreased with increasing speed ($R_{\text{adj}}^{2}$ = 0.092; *p* < 0.001; Figure 2E).

We also assessed the heat distribution as head:hand ratio, head:leg ratio, and leg:hand ratio. We found that there was no effect of sex on these ratios, but the head:leg and leg;hand ratios were significantly related to speed as follows. The head:leg = 1.088–0.006^*^speed, *R*^2^ = 0.066; *p* < 0.001, indicating that the head temperature decreased more with increasing speed than the leg temperature. The increase in the leg:hand ratio with increasing speed (leg:hand = 1.027 + 0.005^*^speed, *R*^2^ = 0.038; *p* < 0.001) indicates that also the hand temperature decreased more with increasing speed than the leg temperature.

## Discussion

The main finding of the present study is that skin temperature of the head, legs, and hands in the final 400 m of a 10,000 m run is lower, the faster the average running speed. This negative association with average speed is more pronounced in the head and hands compared to the legs. The inverse association between average speed and leg skin temperature was more pronounced in women than in men. The lower skin temperature at higher average speed may reflect the impact of airflow that enhances heat loss via evaporation and/or conduction.

### Lower Skin Temperatures With Higher Running Speed

The fact that the faster athletes had the lowest skin temperatures may at least partially be explained by a higher convection and evaporation rate due to the higher wind speed at faster running. In addition, enhanced evaporative heat loss has been reported in trained compared to untrained age-matched people (Stapleton et al., 2015a,b). While the 2°C rise in air temperature during the race may have particularly affected the slower athletes, we consider this effect on the observed skin temperatures to be minor. It has been found that running for 100 min at 60% VO_2_max in 18°C and 31°C resulted in just a 4°C skin temperature difference (Ibrahim et al., 2017) and therefore we expect the skin temperature changes from an ambient temperature change of 2°C to be well below 1°C. Although firm conclusions about whole body heat loss cannot be drawn from the present data, as measures of core temperature are required to substantiate these ideas, overall it appears that increased fitness is associated with more heat dissipation via physical consequences of a higher running speed combined with physiological adaptations.

### High Humidity

The high relative humidity of 88% has probably compromised evaporative cooling. A high humidity may impair human running performance, but temperature appears to have a more pronounced effect on athletic performance (El Helou et al., 2012). In humid conditions, athletes likely had a high skin wetness, decreasing sweating efficiency substantially. Even if younger athletes would have had a higher sweating rate, this would not have made a great difference in these humid conditions. A ceiling effect in evaporative cooling might therefore have blurred a potential age effect. Hyperhidrosis and sweat dripping may have had a substantial effect on individual skin temperatures, but were not assessed by measurements within this study. These factors should be considered in future studies if possible.

### Changes With Age

While age came out as a main effect on skin temperature, the effect was probably more related to the age-related slowing, as speed came out as the main predictor in the model. Indeed, except for a small contribution to head temperature (3%), in none of the stepwise regressions of skin temperature age appeared as a contributing factor. Previous studies also found only a mild effect of age on skin temperature in athletes (Stapleton et al., 2015a,b). Several studies reported decrements in sweating and vascular responses in old athletes (Kenney and Fowler, 1988; Armstrong and Kenney, 1993; Inoue and Shibasaki, 1996; Holowatz et al., 2010; McGinn et al., 2017). The athletes in the latter studies were usually not as well-trained as the sample in our study, which supports the suggestion that older people should engage in regular exercise to improve their thermoregulation and decrease the risk of hyperthermia-related health problems. This is further supported by the observation that none of the athletes 70 years and older had to prematurely terminate the race, while some younger athletes did not finish. Further studies involving measurements of the body core temperature are required to fully evaluate the heat dissipation in younger *vs*. older athletes. In addition, it is possible that two thermo-effector deficiencies cancel each other out, such as reduced cutaneous vasodilation that would reduce skin temperature and a reduction in sweating that would increase skin temperature. Further studies should therefore assess changes in microcirculation and sweating to address this potential aspect in master athletes.

### Sex Differences

In contrast to our hypothesis, the inverse relationship between average speed during the race and leg skin temperature was more pronounced in women than men. One explanation for this difference might be a thicker subcutaneous fat layer in women, where conduction of heat from the underlying muscles to the surface is impaired due to the insulating effect of the subcutaneous adipose tissue. In line with this suggestion is the observation that a greater body fat percentage and a greater skinfold thickness are associated with delayed and lower increases in skin temperature after resistance exercise (Weigert et al., 2018). Even though the subcutaneous fat layer may impair the conductive heat loss from the working leg muscles in women, McGinn et al. reported in 2017 that greater body fat had a minor negative effect on WBHL (McGinn et al., 2017). The maintained heat loss may be realized by an enhanced skin blood flow, evaporation and/or compensatory higher head and hand temperatures for extra heat dissipation. The absence of a higher head and/or hand skin temperature in women than man was, however, indicative that such a sex-difference in redistribution of flow did not occur. Another factor causing warmer legs in men might be an increased muscular heat production as men were on average running at higher speeds. It thus remains to be seen what underlies the lower leg skin temperatures in women compared to age- and performance-matched men.

### Climate Acclimation

Another factor known to affect running performance is acclimation (Périard et al., 2017). Despite age-related changes in microcirculation, older people are able to adapt to new climatic conditions (Kenney and Fowler, 1988; McGinn et al., 2017). As the climate in Malaga was mild with temperatures between 21 and 23°C, acclimation was most likely not a great issue for the athletes. It would, however, be relevant to see whether running in a hot climate will expose differences between sexes and between young-adult and older athletes.

### Limitations, Strength, and Practical Applications

The main limitations of the present study are a lack of baseline data and that no body core-temperatures could be measured. The large number of athletes included in the dataset is the main strength. In addition, the application of infrared skin temperature data collection by thermography in the frame of a running competition is new and shows applicability. It could be a valuable tool for coaches to monitor the extent of heat acclimation to decide about the application of further heat training interventions. Thermography could also be used in extreme sports events such as ultramarathons to improve overall performance and adapt strategies.

## Conclusions

We found no evidence for impaired heat dissipation in the older athletes, further supported by the observation that all starters 70 years and older finished the race. Core temperature measurements, however, are required to study this further. Irrespective of age, a higher average running speed seems to be associated with lower skin temperatures during the last 400 m of a 10,000 m race. The negative relationship between skin temperature and speed was more pronounced in the head and hands than the legs. Interestingly, the leg skin temperature of the women was lower than that of age- and performance-matched men. Core temperature measurements, however, are required to study the potential sex- and age-related differences in thermoregulation further and it remains to be seen whether older athletes are as able to cope with temperature stress as young athletes when racing at high environmental temperatures.

## Data Availability Statement

The datasets generated for this study are available on request to the corresponding author.

## Ethics Statement

The studies involving human participants were reviewed and approved by the Institutional Review Board of RWTH Aachen University Hospital, Pauwelsstr. 30, 52074 Aachen, Germany, approved on October 11, 2017 (reference number EK 300/17). Written informed consent for participation was not required for this study in accordance with the national legislation and the institutional requirements.

## Author Contributions

BG designed the study, collected the data, wrote and approved the manuscript, and generated the figures. HD conducted the statistical analysis, interpreted the data, and corrected and approved the manuscript.

### Conflict of Interest

The authors declare that the research was conducted in the absence of any commercial or financial relationships that could be construed as a potential conflict of interest.

## Footnotes

^1^https://www.timeanddate.com/weather/spain/malaga/historic?month=9&year=2018.

^2^https://drive.google.com/file/d/18r90TSA54rrJSelxsmhanuSOow8IMVp-/view
